# Enhanced Production of Antimicrobial Pyocyanin Using Electromagnetic Fields and Carbon Nanotubes

**DOI:** 10.2147/NSA.S563685

**Published:** 2026-01-12

**Authors:** Joanna Honselmann genannt Humme, Kamila Dubrowska, Dawid Sołoducha, Tomasz Borowski, Adrian Augustyniak, Rafał Rakoczy

**Affiliations:** 1 Department of Chemical and Process Engineering, Faculty of Chemical Technology and Engineering, West Pomeranian University of Technology in Szczecin, Szczecin, Poland; 2 Center for Advanced Materials and Manufacturing Process Engineering (MATPRO), Szczecin, Poland

**Keywords:** antibacterial activity, nanomaterials, phenazines, *Pseudomonas aeruginosa*, stimulation

## Abstract

**Introduction:**

*Pseudomonas aeruginosa* produces pyocyanin, a phenazine antimicrobial agent against drug-resistant microorganisms. Multi-walled carbon nanotubes (MWCNTs) were shown to stimulate pyocyanin production. Since they are known for their conductivity, their stimulatory properties could be affected by electromagnetic fields (EMFs). Therefore, this study aimed to verify whether EMFs, alone or in combination with MWCNT, could serve as a process simulator for pyocyanin production, and whether the production process is optimizable.

**Materials and Methods:**

The Design of Experiment method was employed to optimize pyocyanin production by the cultures exposed to different types of EMFs alone or in combination with MWCNTs. This allowed for identifying the setup with the highest improvement in pyocyanin production. In this setup, additional assays, including conductivity, magnetic induction, ROS level, and membrane potential measurements, were performed. The antibacterial properties of the purified pigment were also assessed.

**Results and Discussion:**

The rotating magnetic field (RMF) combined with MWCNT was identified as the most effective setup for pyocyanin production (production improved by 143% compared to the control), which can be further enhanced by aeration. Significant changes in conductivity, magnetic induction, membrane potential, and ROS levels were observed. The purified pigment exhibited strong antibacterial properties, particularly against *Staphylococcus aureus* and *Acinetobacter baumannii*, which are often recognized as drug-resistant microorganisms.

**Conclusion:**

This research proposes a novel approach to bioprocessing, where the production of the desired metabolite can be stimulated through a combination of stressors.

## Plain Language Summary

Bacteria can produce many useful substances that are used in technology and medicine. One example is pyocyanin, a blue pigment that can kill bacteria. This study looked at whether different types of electromagnetic fields (EMF), alone or combined with tiny tubes called multi-walled carbon nanotubes (MWCNT), could help produce more pyocyanin.

The researchers found that using a rotating magnetic field (RMF) with MWCNT was the best way to produce pyocyanin. Adding air made it even better. They noticed changes in the bacteria’s environment, like conductivity, intensity of the magnetic field, and levels of reactive oxygen species (ROS). The bacteria’s cell membranes also reacted differently to the RMF and MWCNT.

The purified pyocyanin was highly effective at killing or inhibiting the growth of bacteria, especially *Staphylococcus aureus* and *Acinetobacter baumannii*, which are often drug-resistant. This research suggests a new way to produce useful substances by combining different stress factors.

## Graphical Abstract

**Figure:**
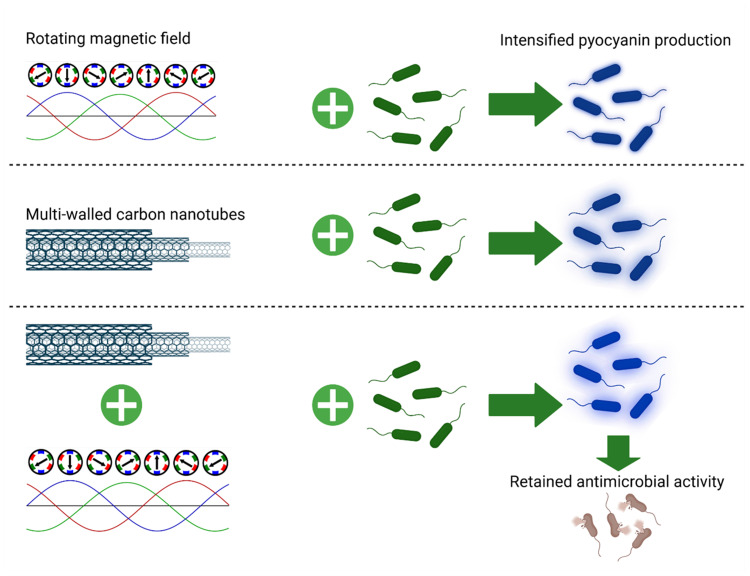

## Introduction

Electromagnetic fields (EMFs) can be considered as non-conventional bioprocess enhancers. So far, EMFs have been used to increase the growth rate of the cultures, performance of wastewater treatment process, biogas production, water purification, biofilm reduction, degradation of toxic chemicals, and biomass and metabolite production.^1^ Various mechanisms have been assigned to the effects caused by EMFs, with the most probable being the shift in the transport of substances between the cells and their environment by modifying the membrane potential.^1–3^ However, despite numerous observed effects, the mechanisms remain unclear.

Despite that, the recent approach combines EMFs with other bioprocess-enhancing factors, i.e., antibiotics,^4^ essential oils,^5–7^ or nanomaterials.^8^,^9^ While considered separately, these factors are known for altering the physiology of bacterial cells. Previous works have proven that EMFs influence the growth rate, viability, and pyocyanin (PYO) production by *Pseudomonas aeruginosa*.^10^,^11^ Similarly, nanomaterials, eg, ZnO nanoparticles and multi-walled carbon nanotubes (MWCNTs), have also been identified as boosters for the quantity of produced PYO.^12^,^13^ Particularly, MWCNTs that proved to be a strong stimulator of pyocyanin production are known for their conductivity, which could facilitate changes in the environment leading to shifts in redox balance, membrane potential, or electron transfer, if an electromagnetic field was present.^14^ So far, no studies have shown how these two factors (MWCNTs and EMFs) will affect the metabolite production in *P. aeruginosa*.

Pyocyanin is a bacterial phenazine produced by *P. aeruginosa* and *P. paraeruginosa*, known for its blue color, which is a significant virulence factor in nosocomial infections caused by these microorganisms. On the other hand, it has been gaining increasing interest due to its unique antimicrobial and redox properties that can be utilized in many branches of technology and medicine.^15^ The antimicrobial potential of pyocyanin is particularly significant in the era of antibiotic resistance, as the newest reports focused on antimicrobial resistance (AMR) estimated that in 2050, 1.91 million deaths could be attributable to AMR, and 8.22 million could be associated with it.^16^

To be used in industry, PYO needs to be produced in higher quantities. Various methods have been employed to enhance its production, including stimulation by O_2_ scavengers such as n-hexane.^17^ MWCNT also has considerable potential in stimulating its production. However, these nanomaterials have never been used in conjunction with EMFs, and no optimization protocols have been established for EMFs alone or in combination with MWCNT. Therefore, it is unclear whether these factors can be optimized to positively influence PYO production in *P. aeruginosa*.

For this reason, this work aimed to optimize pyocyanin production using EMFs or EMFs combined with a nanomaterial, employing the Design of Experiment methodology, and to monitor bacterial physiology under optimal conditions to elucidate the probable mechanism. Finally, the antimicrobial properties of pyocyanin obtained in optimal conditions were assessed.

## Materials and Methods

### Material

The bacterial strain used in this research was *Pseudomonas aeruginosa* ATCC 27853, a recognized producer of pyocyanin. The cultivations were conducted in 10 mL of King’s A medium for 72 hours. The experiments were carried out using EMF-assisted reactors constructed in our laboratory. Their characterization was described in detail in our previous works.^10^,^18^ In this research, the tested types of EMFs were a rotating magnetic field (RMF), a static magnetic field with positive polarity (SMF+), and a static magnetic field with negative polarity (SMF−). After the experiments, all liquid material was autoclaved, and disposables (including nanomaterials) were removed to proper containers in accordance with the standard for a second biosafety level (BSL2).

### The Process Optimization

#### Exposure to Electromagnetic Field

**Figure 1:**
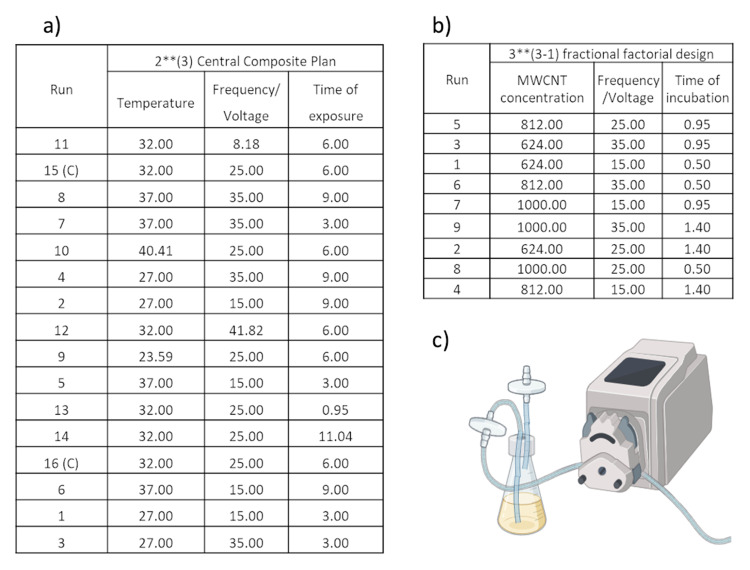
The plan of experiments: (**a**) pyocyanin production under exposure to EMFs; (**b**) pyocyanin production under exposure to EMFs and MWCNT; (**c**) setup for the aeration assay; the description 3**(3–1) indicates that a 1/3 fraction of the full 3^3^ design was applied in planning.

The plan of experiments was created for each setup (RMF, SMF+, and SMF−) employing Statistica software (version 14.0.0.15, TIBCO Software Inc., USA). The method applied was the Design of Experiments with a central composite plan. In the case of the rotating electromagnetic field, the input parameters were temperature, exposure time, and the frequency of the electromagnetic field. On the other hand, the input parameters for the static electromagnetic field were temperature, exposure time, and voltage. The created plans are presented in Figure 1a. Based on the obtained results, the significant factors were identified, surfaces were fitted, and the optimal conditions were calculated.

#### Exposure to Electromagnetic Fields and Nanomaterials

The method applied was the Design of Experiments with a 3**(3–1) fractional factorial design. The input factors were the nanomaterial concentration, the frequency or voltage of the EMF, and the time of exposure. The created plan is presented in Figure 1b. Based on the obtained results, the significant factors were identified, surfaces were fitted, and the optimal conditions were calculated.

### Production and Extraction of Pyocyanin

The pyocyanin-producing microorganism used in this study was *Pseudomonas aeruginosa* ATCC 27853. Shortly, the strain was kept at −20°C on MicroBank and was revived on TSA medium at 37°C. Before the experiment, the fresh medium was inoculated with bacteria for an overnight culture in TSB medium (37°C, 100 rpm). After incubation, the optical density (absorbance at λ = 600 nm) of the culture was adjusted each time to 0.5, and King’s A broth was inoculated (1:100 v/v) for further tests. The experiments were conducted in 50 mL Falcon-type tubes with caps equipped with filters. The volume of the culture equaled 10 mL. After the exposure time indicated by the plan, the cultures from the Falcon-type tubes were transferred to a 90 mm Petri dish and incubated until they reached 72 hours of the total culture time. After that, the cultures were centrifuged, and the supernatant was subjected to pyocyanin extraction with chloroform and re-extraction with 0.2 M HCl. The concentration of the extracted pyocyanin was measured spectrophotometrically at 520 nm, calculated from the calibration curve and expressed as μg of pyocyanin per mL of the culture.^13^ Spectrophotometric analysis (UV-Vis absorption spectrum) of the product was performed to compare products obtained in production variants (Supplementary material, Figure S1).

### The Influence of Aeration on Pigment Production

Aeration was applied to determine whether the optimal setup could be further optimized. The samples were incubated at the optimal process parameters and later transferred to Erlenmeyer flasks equipped with a plug with two tubes ended with 0.22 µm filters (one filtering the air coming in and one filtering the air coming out, as shown in Figure 1c). The flow rate was set to 2 mL/min using the 4-channel LeadFluid BT300S peristaltic pump (Aqua-Trend, Łódź, Poland), which resulted in a steady aeration of the culture without creating unwanted foaming or cell disturbance. The incubation period lasted 72 hours, and the cultures were subsequently subjected to chloroform-HCl extraction.

### Measurements of the Process Parameters

To determine how the RMF+MWCNT system operates, three process parameters, such as electrical conductivity, magnetic induction, and oxygenation, were measured. The first parameter was measured using a conductivity probe (ECF-1) and a multifunction meter (CX-601, ELMETRON, Zabrze, Poland). The measurement procedure started with placing Falcon-type tubes with and without MWCNT under the exposure of RMF. RMF-treated and control samples during the experiment were maintained in thermostatic conditions at 32°C ± 0.1°C. After one hour in each sample, electrical conductivity was measured by flooding the probe in a Falcon-type tube for 20 seconds, sampling every second. The measurement procedure for magnetic induction was similar; RMF and control samples were placed in Falcon-type tubes and left for an hour. After that time, magnetic induction was measured for 20 seconds, with sampling every second, using a gaussmeter (GM-2) with a radial probe. The last parameter required a more complex procedure, which took the same amount of time as the previous parameters. After this, the oxygen probe was left inside the Falcon-type tube for one minute. For measurements, a COG-1 oxygen probe (ELMETRON, Zabrze, Poland) was used. After the required waiting time (one minute), the probe was coupled with CX-601, and measurements were recorded for 20 seconds with a sampling interval of one second.

### Characterization of the Optimized Setup

The generation of OH· radicals was assessed using terephthalic acid (TA) methodology.^19^ Shortly, terephthalic acid was mixed with the King’s A medium containing MWCNTs or with no MWCNT addition. The samples were placed in the reactors and exposed to RMF for 30 minutes. After that, the fluorescence of the samples was measured at λ_ex_= 315 nm and λ_ex_= 430 nm.

The reactive oxygen species in the cells were assessed using DCFH-DA according to the user’s manual. Shortly, the cells were incubated in the reactors, and later DCFH-DA was added (final concentration of 100 µM) and incubated for 1.5 hours in the dark. The fluorescence was measured at ʎ_ex_= 485 nm and ʎ_ex_= 530 nm.

Membrane potential and the viability (LIVE/DEAD assay) of the control cells and the cells exposed to RMF+MWCNT were assessed after 24 hours on flow cytometry according to the previously described methodology.^13^ Studies were performed on the BD Accuri C6 Plus cytometer. Ten thousand events were recorded in each case.

### Antimicrobial Properties of Pyocyanin

To assess the antimicrobial properties of the purified pyocyanin (purification steps were in line with the procedure described by Honselmann et al^14^), we conducted analyses of optical density and aerobic respiration (based on resazurin reduction, which can also be interpreted as metabolic activity) after 24 hours. The microbial models chosen for the assay were *Escherichia coli* ATCC 8739, *Klebsiella pneumoniae* BAA-1706, *Acinetobacter baumannii* ATCC 19606, *Staphylococcus aureus* ATCC 33591, and *Candida albicans* ATCC 10231. The bacterial cultures were cultivated overnight in TSB medium at 37°C. Next, bacteria were inoculated into fresh TSB with varying pyocyanin concentrations (2-fold dilutions from 500.00 to 3.91 µg/mL; pyocyanin was in its native blue form; pyocyanin stock was dissolved in a mixture of 10% ethanol and 90% deionized, sterile water). The assay was conducted in a 96-well polystyrene plate, excluding all the edge wells to avoid evaporation. The plate was incubated at 37°C for 24 hours. After that, the optical density (λ = 600 nm) and the fluorescence (λ = 520/590 nm) of the cells were measured. The fluorescence reads were based on the resazurin reduction by the respirating cells (detailed description was provided by Augustyniak et al^20^). The control plate counts were performed to verify the obtained results. The positive control included bacteria in King’s A medium, with the addition of the ethanol/water mixture (without pyocyanin), and the negative control consisted of King’s A medium with the addition of pyocyanin dissolved in the ethanol/water mixture (without bacteria).

### Statistical Analysis

Statistical analyses were performed in OriginLab software using one-way ANOVA analysis, with Tukey’s test for mean comparison, at p < 0.05. All optimization experiments were conducted in 3 replicates, and the aeration assay in 4 replicates. Conductivity, magnetic induction, dissolved oxygen content, membrane potential, LIVE/DEAD assay, and ROS generation with terephthalic acid and DCFH-DA were measured in 3 replicates. Antimicrobial properties were assessed for 6 (OD measurements) and 5 replicates (resazurin reduction assay) to increase the statistical power and ensure alignment with the study objectives.

## Results and Discussion

### The Process Optimization

#### Electromagnetic Field Exposure

**Figure 2:**
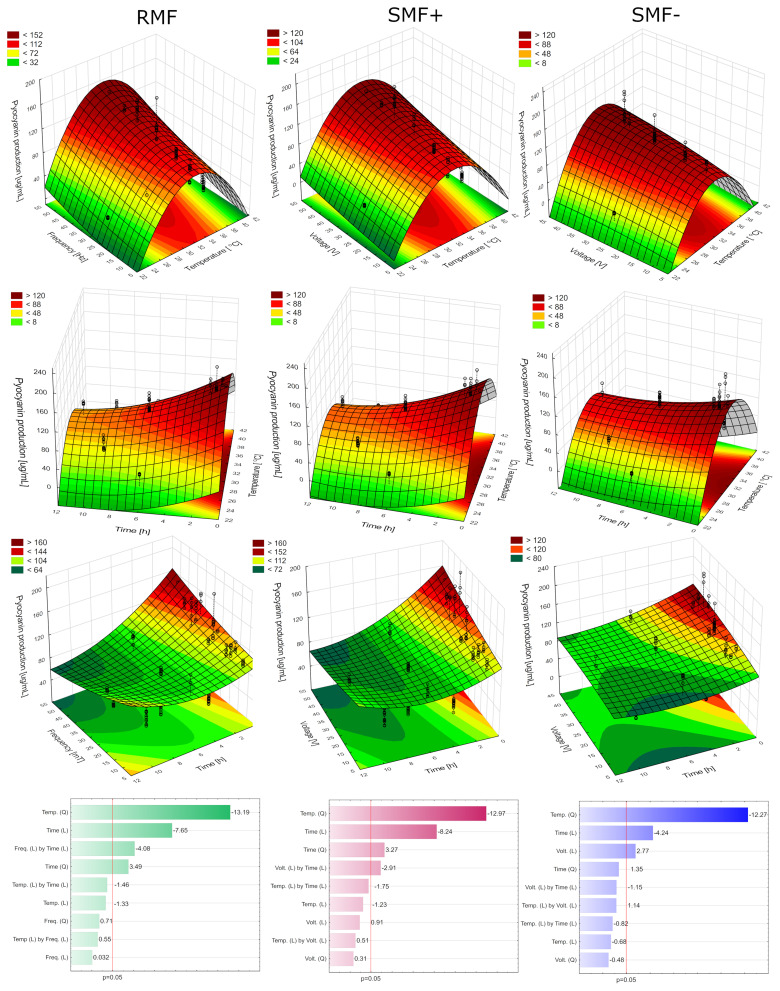
Optimization of pyocyanin production with the use of RMF, SMF+, and SMF−.

The execution of DoE plans indicated which process conditions significantly influenced the production of pyocyanin (Figure 2). In the case of RMF (R^2^= 0.80), the production of the pigment was significantly influenced by the temperature (quadratic), time (linear and quadratic), and the interaction of the frequency and the time. The optimal conditions calculated by the software were 32°C, 50 Hz, and 15 minutes of exposure. The experimental results at the optimal conditions resulted in improved pyocyanin by 11% (relative to the control). In the case of SMF+ (R^2^= 0.80), the production of the pigment was also significantly influenced by the temperature (quadratic), time (linear and quadratic), and the interaction of the voltage and the time. The optimal calculated conditions were 32°C, 50 Hz, and 15 minutes of exposure, which resulted in 9% higher pyocyanin production compared to the unexposed culture. In the case of SMF− (R^2^= 0.72), the production of the pigment was significantly influenced only by the temperature (quadratic), time (linear), and voltage (linear). The optimal conditions calculated by the software were 32°C, 41.82 Hz, and 15 minutes of exposure, resulting in only a 3% improvement in pyocyanin production compared to the control. Overall, RMF turned out to be the most effective electromagnetic stimulator of pigment production. However, the stimulation obtained only with EMF was relatively low, which was likely due to the relatively short incubation time. This was confirmed in another study, where longer (6-hour) incubation under SMF and RMF reached around 24–55%.^10^ Nevertheless, our results confirmed that even short exposure to EMF can alter phenazine production in *P. aeruginosa*.

#### Electromagnetic Field and Nanomaterials Exposure

**Figure 3:**
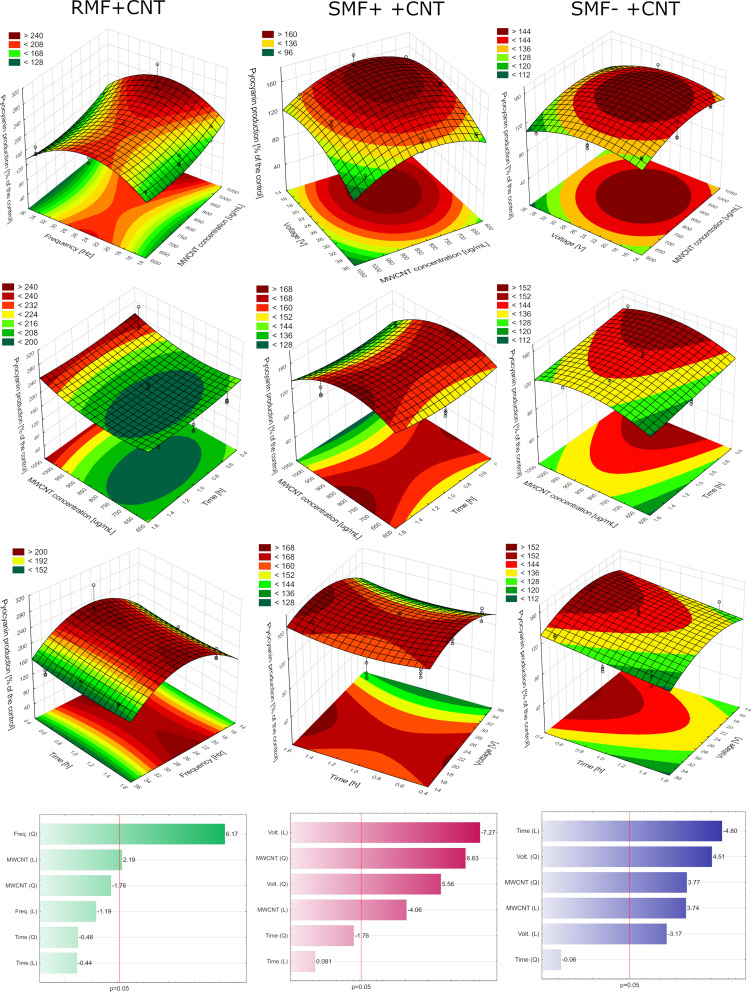
Optimization of pyocyanin production with the use of RMF+MWCNT, SMF+ + MWCNT and SMF− + MWCNT.

In the case of RMF+MWCNT (R^2^= 0.70), the production of the pigment was significantly influenced only by the frequency (quadratic) and the MWCNT concentration (linear) (Figure 3). The optimal conditions calculated by the software were 1000 µg/mL, 25 Hz, and 30 minutes of exposure. The optimized culture produced 143% more pyocyanin than the control. In the case of SMF+ + MWCNT (R^2^= 0.88), the production of the pigment was significantly influenced by the voltage (linear and quadratic) and the MWCNT concentration (quadratic and linear). The optimal conditions calculated by the software were 812 µg/mL (MWCNT), 20 Hz, and 1:24 hours of exposure. The optimization resulted in a 35% increase in pyocyanin production compared to the control. In the case of SMF− + MWCNT (R^2^= 0.80), the production of the pigment was significantly influenced by the voltage (linear and quadratic), the MWCNT concentration (quadratic and linear), and the time (linear). The optimal conditions calculated by the software were 906 µg/mL, 25 Hz, and 30 minutes of exposure. Pyocyanin production was enhanced by 34% compared to the control.

Overall, EMFs alone exhibited the lowest stimulative activity, while the combination of EMFs and MWCNT proved to be more effective. Based on the results obtained, the setup with RMF+MWCNT was chosen for further analyses as it exhibited the highest stimulation results. A scale-up of pyocyanin production in such a setup could be challenging due to the limited capacity of the reactor. However, a short incubation time of the culture could make it plausible, as the whole production process does not need to be conducted in a magnetically assisted reactor. The developed co-stimulated process provided a novel alternative for efficient pyocyanin production compared to other methods based on chemical additives^17^ or genetically engineered *E. coli*.^21^

### The Influence of Aeration on Pigment Production

**Figure 4:**
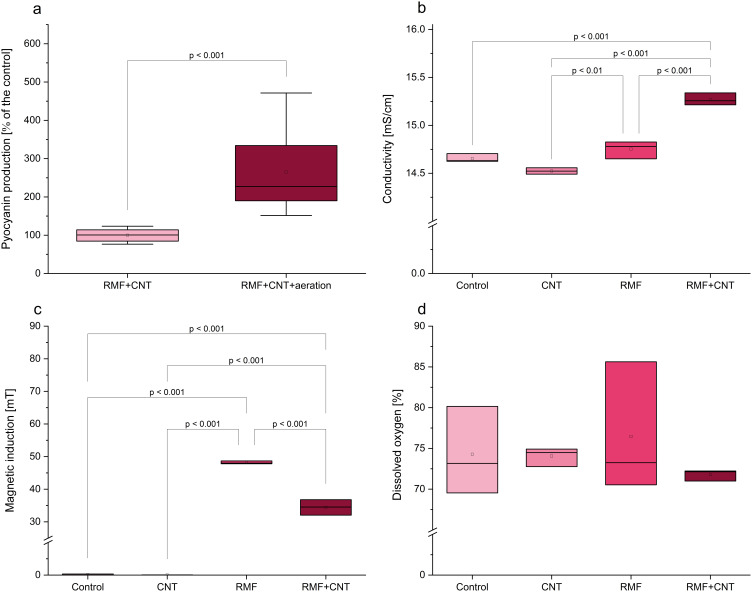
The changes in (**a**) pyocyanin production in the presence of aeration or without, and (**b**) conductivity, (**c**) magnetic induction, (**d**) dissolved oxygen in the presence of MWCNT, RMF, or both factors.

The aeration of the optimized culture increased pyocyanin production by more than 150% in comparison to the control (Figure 4d). This confirms that the process can be further improved by the addition of aeration to the culture. Oxygen is necessary to convert *phz*M product to pyocyanin by 5-methylphenazine-1-carboxylate 1-monooxygenase coded by *phz*S gene. Oxygen content also regulates the PQS system, which is associated with the regulation of operons coding for phenazine biosynthesis.^22^

### Measurements of the Process Parameters

For a deeper understanding of the RMF+MWCNT mode of action, factors like sample conductivity, magnetic induction, and dissolved oxygen content were investigated. The analysis of the samples’ conductivity showed a significantly higher value in the RMF+MWCNT sample when compared to the control (Figure 4a). In the case of the samples with RMF (both RMF alone and RMF+MWCNT), the conductivity was significantly higher than in the MWCNT sample. The magnetic induction in the sample RMF was significantly higher than in the control, MWCNT, or RMF+MWCNT (Figure 4b). This suggests that MWCNT lowers the magnetic induction in the setup. The dissolved oxygen percentage was the highest in the RMF sample (Figure 4c). However, no differences were statistically significant. Based on the information gained, we assume that the addition of MWCNT provides an enhanced environment for the cells that is characterized by higher conductivity, although lower magnetic induction. There is scarce literature that may explain the observed stimulation of pyocyanin production under these conditions. Raouia et al have shown that higher magnetic induction may increase swarming motility in an exposed wild strain of *P. aeruginosa*.^11^ Since this bacterium produces more pyocyanin in the settled state, this parameter could be the contributing factor for obtaining better results in the combination of factors than in EMF alone. Pyocyanin in microcolonies and biofilms serves as an electron shuttle, transporting electrons to the oxygen-limited biofilm interior, thereby creating a redox loop where the molecule shifts between its oxidized and reduced states.^23^ It is a form of electrochemical communication between cells of *P. aeruginosa* that may be, by its nature, altered by increased conductivity.

### Characterization of the Optimized Setup

**Figure 5:**
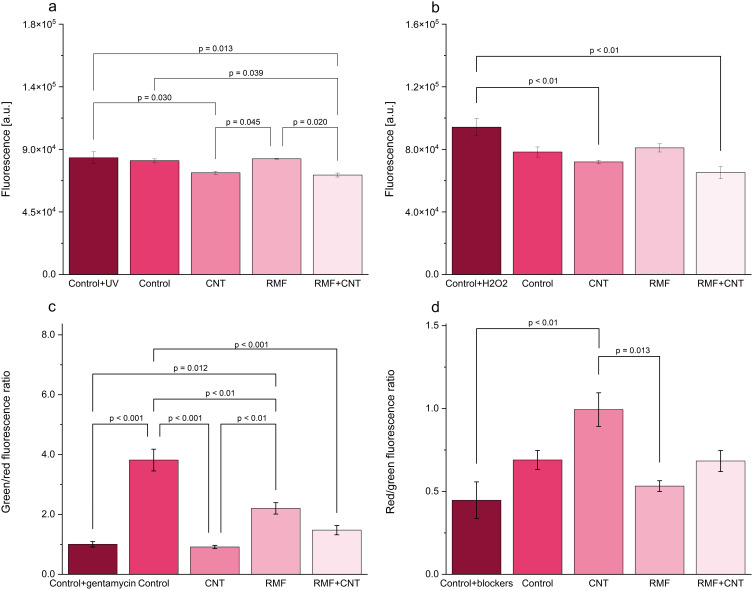
The changes in (**a**) OH· generation, (**b**) intracellular ROS levels, (**c**) LIVE/DEAD assay, and (**d**) membrane potential assay.

The possibility of ROS generation by CNTs has been mentioned in the literature.^24^ Therefore, the ROS generation was monitored in both setups, with and without bacteria. The results of OH· generation in the setup without bacteria showed significant differences (Figure 5a). The lowest fluorescence was observed for the sample RMF+MWCNT, which was significantly lower than that of the control+UV (positive control), control, and RMF samples. Moreover, the MWCNT sample exhibited the second lowest fluorescence, which was significantly different from the RMF samples, which had the highest fluorescent signal among non-UV-exposed samples. This indicates that RMF exposure leads to OH· generation. However, this increase is relatively low and not statistically significant compared to the control. The assay of the intracellular ROS level (Figure 5b) showed similar findings to the terephthalic acid assay. Once again, the lowest fluorescence level was detected in the RMF+MWCNT sample, followed by the MWCNT sample (both significantly lower than the H_2_O_2_-spiked positive control). RMF fluorescence was the highest among the non-spiked samples, indicating that RMF exposure is associated with higher ROS levels within the cells. Both ROS-related assays demonstrated that the addition of MWCNTs reduced the detected ROS levels. Interestingly, even though RMF exposure alone had higher ROS reads, when applied with MWCNTs, it resulted in the lowest ROS signals. This could be attributed to the ROS-scavenging activity of CNTs that has been described in the literature.^25–30^ Fenoglio et al^29^ demonstrated that MWCNTs can act as scavengers of hydroxyl and superoxide radicals, potentially providing protective properties against oxidative stress in bacterial cells.

LIVE/DEAD assay (Figure 5c) allowed the assessment of the ratio of living (green fluorescence intensity) to dead cells (red fluorescence intensity). The results have shown that after 24 hours of culturing, the ratio significantly drops in all the tested setups when compared to the control culture. Interestingly, the most pronounced drop was observed in the MWCNT sample, which was comparable to the control with gentamycin (a control with the antibiotic used to track the dead population). However, the sample RMF+MWCNT scored a ratio value higher than the one for the MWCNT sample and lower than the one reported for the RMF sample. Nevertheless, we have previously observed that the viability of the cells incubated with MWCNT was higher than in the control up to 24 hours, and after this time it significantly dropped.^14^ Membrane potential assay (Figure 5d) showed an elevated ratio of red to green fluorescence in the case of the MWCNT sample. This may indicate an increased membrane transport compared to the control culture. The RMF sample was characterized with a lower ratio, but the RMF+CNT sample’s ratio had an intermediate value between the MWCNT and RMF sample values. The obtained results might indicate a higher membrane potential and a lower LIVE/DEAD ratio in the case of the MWCNT sample. However, it was previously reported that cells with a high membrane potential increasingly uptake propidium iodide (red stain in the LIVE/DEAD assay) and can be falsely identified as dead cells.^31^ A lower LIVE/DEAD ratio in the MWCNT sample, combined with a higher membrane potential, suggests that this may be the case in the current study. Interestingly, the combination of MWCNT and RMF alleviated this effect.

### Antimicrobial Properties of Pyocyanin

**Figure 6:**
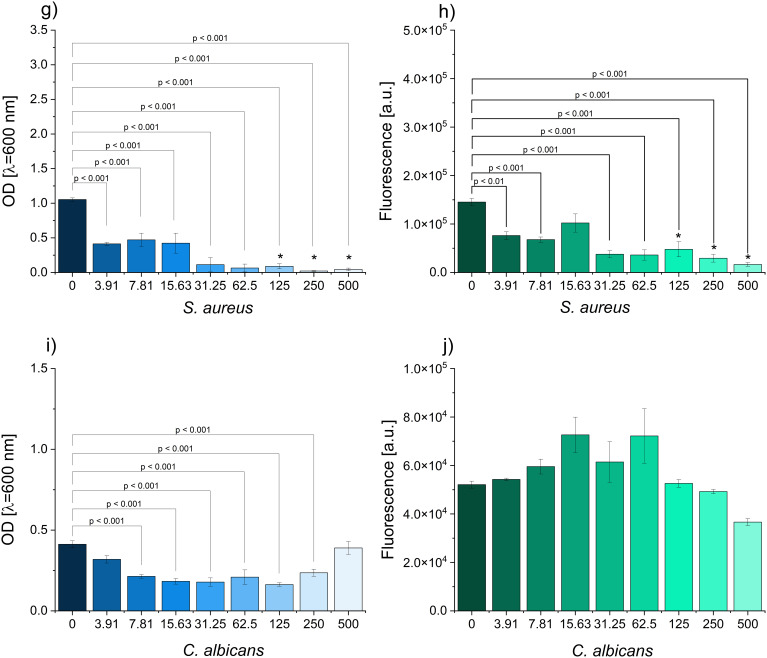
The influence of pyocyanin on the optical density (left) and metabolic activity (right) of (**a-b**) *E. coli*, (**c-d**) *K. pneumoniae*, (**e-f**) *A. baumannnii*, (**g-h**) *S. aureus* (**i-j**) *C. albicans* (*indicates no growth in plate count assay).

The monitoring of the OD and resazurin reduction allowed for assessing the antimicrobial properties of pyocyanin. In the case of *E. coli*, monitoring of the optical density (OD) showed a significant reduction starting from 125 µg/mL (Figure 6a). At the concentration of 500 µg/mL, no growth was observed in either the microplate or the plate count assay. Interestingly, metabolic respiration was significantly lowered at the concentration of 31.25 µg/mL and higher. The same concentrations as for *E. coli* were reported for *K. pneumoniae* (i.e., 125 and 500 µg/mL) (Figure 6b). However, interesting results were noted in the resazurin reduction assay. The lowest concentrations of pyocyanin added to the culture resulted in the stimulation of aerobic respiration, while the higher ones significantly reduced it. This suggests that sublethal concentrations of pyocyanin can affect the metabolic activity of bacteria in response to stress. In the case of *A. baumanniii*, the OD was significantly lowered at 7.81 µg/mL of pyocyanin concentration (Figure 6c). On the other hand, the bactericidal effect was reported for the concentration of 31.25 µg/mL. Both concentrations are much lower than the ones reported for other Gram-negative bacteria (*E. coli* and *K. pneumoniae*). A similar trend was observed in the resazurin reduction assay, as concentrations of pyocyanin 15.63 µg/mL or higher led to significantly decreased fluorescence readings. In the case of *S. aureus*, the lowest tested pyocyanin concentration, 3.91 µg/mL, significantly inhibited the OD. At the same time, the bactericidal effect was reached for the concentration of 125 µg/mL (Figure 6d). It was also observed that the aerobic respiration was significantly lowered in all the cases except for the concentration of 15.63 µg/mL. *C. albicans* was characterized by the reduced OD starting at 7.81 µg/mL (higher reads were obtained for 500 µg/mL) (Figure 6e). Moreover, no fungicidal effect was observed for this microorganism. The resazurin assay showed increased fluorescence reads within the middle range of the tested concentrations and a drop at the highest ones. However, none of the differences were significant at p ≤ 0.05.
**Figure 6:**
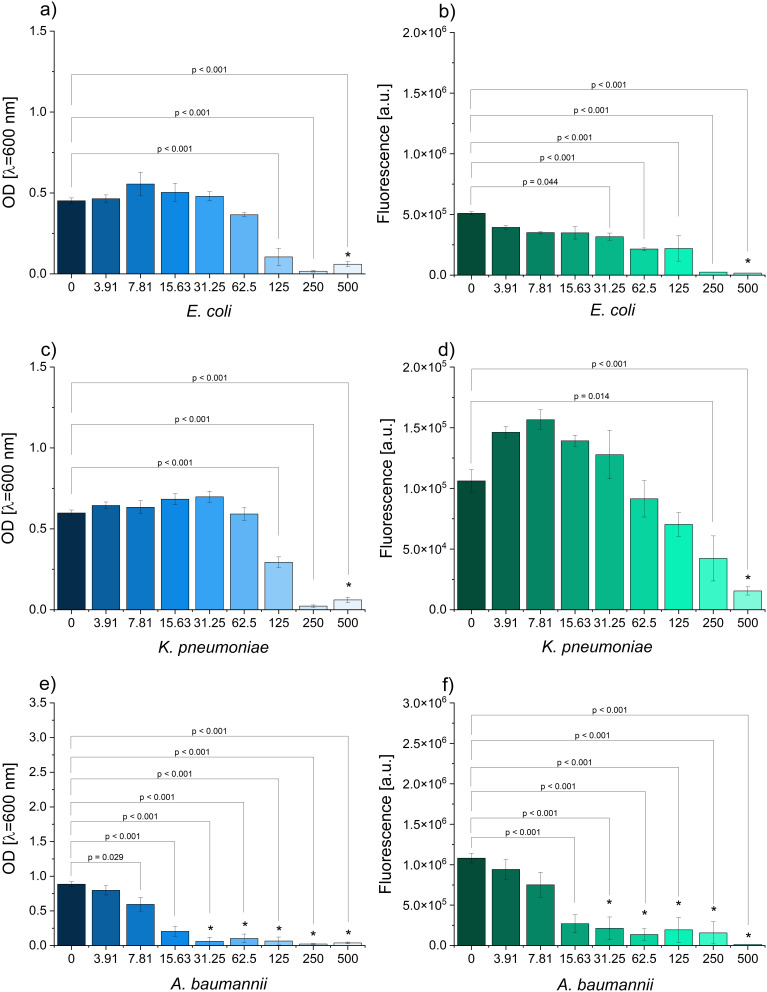
Continued.

The results showed that pyocyanin was most effective against *A. baumannii* and *S. aureus*. Both microorganisms belong to the ESKAPE group and are frequently reported as multidrug-resistant.^32^ These studies confirmed the previously reported antibacterial potential of pyocyanin and that the pigment produced with the optimized method retained its activity. The use of the extracted product is vital, because *P. aeruginosa* produces a variety of factors that can be antibacterial, that apart from pyocyanin, includes siderophores, rhamnolipids, quinolones, and other phenazines.^33^

## Conclusion

We have demonstrated for the first time that the combination of electromagnetic fields and nanomaterials can be utilized to optimize pyocyanin production. Moreover, DoE methods could be successfully used for this purpose. RMF can interact with MWCNT, causing specific changes in the physicochemical conditions of bacterial culture through alterations in conductivity and magnetic induction of the culture environment. The influence of these factors on *P. aeruginosa* was not neutral to the bacterial population. Their activity could cause contradictory responses in the viability and membrane potential of participating cells. However, these effects do not prevent the bacterium from producing a functional metabolite. The produced pyocyanin retained its color and antimicrobial activity, which is crucial for its further application as an antibacterial agent. Our study has also shown that even short incubation of culture under EMF may induce lasting changes in pigment production in *P. aeruginosa*, which was also reported for the first time.
